# Genomic analyses of Asiatic Mouflon in Iran provide insights into the domestication and evolution of sheep

**DOI:** 10.1186/s12711-025-00978-y

**Published:** 2025-06-13

**Authors:** Dong-Feng Wang, Pablo Orozco-terWengel, Hosein Salehian-Dehkordi, Ali Esmailizadeh, Feng-Hua Lv

**Affiliations:** 1 Frontiers Science Center for Molecular Design Breeding (MOE), State Key Laboratory of Animal Biotech Breeding, College of Animal Science and Technology, China Agricultural University, Beijing, 100193 China; 2 CAS Key Laboratory of Animal Ecology and Conservation Biology, Institute of Zoology, Chinese Academy of Sciences (CAS), Beijing, China; 3 College of Life Sciences, University of Chinese Academy of Sciences (UCAS), Beijing, China; 4 School of Biosciences, Cardiff University, Cardiff, UK; 5 Institute of Grassland Research of Chinese Academy of Agricultural Sciences (CAAS), Hohhot, 010010 China; 6 Department of Animal Science, Faculty of Agriculture, Shahid Bahonar University of Kerman, Kerman, 76169-14111 Iran

## Abstract

**Background:**

Asiatic mouflon (*Ovis gmelini*) consists of several subspecies mainly distributed in Armenia, southern Azerbaijan, Cyprus, northern, southern, and western regions of Iran, and eastern and central regions of Turkey nowadays. Genome analyses of Asiatic mouflon in Iran revealed that they could have diverged from the direct ancestor of domestic sheep, and showed genetic introgression into domestic sheep after domestication. However, the impact of the Asiatic mouflon subspecies in Iran on sheep domestication remains unclear.

**Results:**

Here, we conducted a comprehensive population genomics analysis of Asiatic mouflon in Iran with 788 whole-genome sequences (including 40 from Asiatic mouflon), 1104 whole mitogenomes (105 from Asiatic mouflon), and 239 Y chromosomes (21 from Asiatic mouflon). Whole-genome sequence analyses revealed two subpopulations of Asiatic mouflon in Iran: *O. gmelini*_2 limited on Kaboodan Island in Urmia Lake National Park and *O. gmelini*_1 over a wide geographic area. Phylogenetic analyses of Asiatic mouflon in Iran based on uniparental variants revealed a monophyletic lineage with the mitochondrial haplogroups C/E, and clustered into a monophyletic with Y-chromosomal lineage HY2 of sheep. Additionally, introgression tests detected significant signals of genetic introgression from *O. gmelini*_2 to four sheep populations (e.g., Garut, Bangladeshi, Nellore, and Sumatra) in South and Southeast Asia. In the four sheep populations, selective tests and introgression signals revealed that the wild introgression could have contributed to their body size, fat metabolism and local adaptation to the hot and humid environments in the Indian Peninsula.

**Conclusions:**

Our results clarified subpopulation structure of Asiatic mouflon in Iran, identifying two distinct groups: *O. gmelini*_1 and *O. gmelini*_2. Additionally, we suggest a potential genetic contribution to domestic sheep by introgression, with maternal haplogroup C and paternal lineage HY2 likely originating from the Asiatic mouflon populations in Iran. Our findings offer new insights into domestication of sheep and subsequent introgressions events from wild relatives to domestic populations.

**Supplementary Information:**

The online version contains supplementary material available at 10.1186/s12711-025-00978-y.

## Background

Archeozoological and genetic evidences suggested that Asiatic mouflon (*O. gmelini*) have underwent a period of multigenerational human management in southeastern Anatolia by ~ 10,500 years B.P. [1–5]. Following domestication, sheep diffused throughout Eurasia with human migrations and developed into different populations under natural and artificial selections [6–9]. Asiatic mouflon range over a wide geographic area in Armenia, southern Azerbaijan, Cyprus, northern, southern, central and western regions of Iran, and eastern and central regions of Turkey. It has been classified into five subspecies based on morphological differences and geographic distributions [10, 11], such as Armenian mouflon (*O. gmelini gmelini*), Anatolian mouflon (*O. gmelini anatolica*), Isfahan mouflon (*O. gmelini isphahanica*), Laristan mouflon (*O. gmelini laristanica*), and Cyprus mouflon (*O. gmelini ophion*). Due to the complex intraspecies taxonomy of Asiatic mouflon, the genetic contributions of various subspecies to the domestication of sheep remains unclear [4, 12–14].

Previous genetic studies revealed controversial ancestral origins of domestic sheep. Hiendleder et al. proposed that sheep were domesticated from two different subspecies in Turkey and western Iran, corresponding to mtDNA lineages A and B of domestic sheep, respectively [13]. Through genetic and phylogeographic analyses of partial mtDNA sequences of modern and ancient samples in wild and domestic sheep, Demirci et al. suggested that domestic sheep could have originated from two distinct ancestral populations of mtDNA lineages A + B + D and C + E of domestic sheep, respectively [4]. Recent studies, incorporating genetic and geographic evidence from Asiatic mouflon samples across their distribution range, along with modern and ancient sheep samples from Anatolia, Southwest Asia, Europe, and Africa, suggested that early sheep management likely originated in Anatolia and is associated with the Anatolian mouflon subspecies (*O. g. anatolica*) [5, 15–17].

Recently, Y-chromosome and whole-genome analyses revealed that Iranian Asiatic mouflon may have diverged from the direct ancestor of domestic sheep, and showed genetic introgression into domestic sheep post-domestication [9, 14, 18]. Also, these studies have demonstrated that Asiatic mouflon in Iran are crucial for the development of morphological and adaptive characteristics in domestic sheep [9, 18, 19]. Nevertheless, the impact and extent of genetic introgression involving the Asiatic mouflon subspecies in Iran (e.g., *O. gmelini gmelini*, *O. gmelini isphahanica*, and *O. gmelini laristanica*) and their hybrid populations (*O. gmelini gmelini* × *Ovis vignei*) on the genomes of domestic sheep remains unclear.

Here, we comprehensively analyzed whole genome sequence (n_amuf_ = 40; n_total_ = 788), complete (n_amuf_ = 105; n_total_ = 1104) and partial (n_amuf_ = 244; n_total_ = 337) mtDNA sequences, and Y-chromosome genetic variants (n_amuf_ = 21; n_total_ = 239) in domestic sheep and wild sheep, covering a large distribution area in Asia, Europe, Africa, and America. We aimed to elucidate the contributions of various Asiatic mouflon subspecies in Iran to the domestication of sheep. Additionally, we investigated the impact of wild introgression from the Iranian Asiatic mouflon subspecies on the genomes of domestic sheep populations in South and Southeast Asia, which have adapted to the hot and humid environments.

## Methods

### Datasets

Whole-genome sequences (WGS) of 788 individuals representing 158 domestic sheep populations and three wild sheep species [40 Asiatic mouflon (*O. gmelini*), 9 urial (*O. vignei*), and 1 bighorn sheep (*Ovis canadensis*)] were obtained from publicly available data (see Additional file 1: Table S1). Besides these, we retrieved 327 whole mitogenomes from the previous studies (including 105 from Asiatic mouflon) (see Additional file 1: Table S2). Also, we included 337 Cytochrome b (*Cyt-b*) sequences (including 244 from Asiatic mouflon) in the integrated analysis (see Additional file 1: Table S3).

### Variant calling

We implemented quality-based trimming for WGS data with Trimmomatic v0.39 [20] and mapped high-quality reads to the reference genome *Oar_rambouillet_v1.0* and *CM022046.1* (https://www.ncbi.nlm.nih.gov/assembly/GCF_002742125.1/; https://www.ncbi.nlm.nih.gov/nuccore/CM022046.1/, last accessed May 12, 2020) using the Burrows–Wheeler aligner (BWA mem) v.0.7.8 [21] with the default parameters. Then, we removed duplicates with the *MarkDuplicates* module in GATK v 4.1.2.0 [22]. We used the *HaplotypeCaller* module in GATK v 4.1.2.0 to generate the GVCF file of each sample and merged the raw GVCF file of each individual with the *CombineGVCFs* module. Afterward, we genotyped SNPs using the *GenotypeGVCFs* module and filtered SNPs using the *VariantFiltering* module of GATK with the parameters “QUAL < 30.0||QD < 2.0||MQ < 40.0||FS > 60.0||SOR > 3.0||MQRankSum < − 12.5||ReadPosRankSum < − 8.0”. We obtained a total of 105,817,835 high-quality autosomal SNPs and 203,938 Y-chromosomal SNPs for the following analysis.

### Mitochondrial DNA assembly

We calculated the mitogenome depth of each sample using the SAMtools v.1.9 [23] and kept only the samples with depth > 100 in the analyses below. We assembled the complete mitogenomes using the following protocol. We first converted BAM files to the fastq format. Then, we generated the mitogenome consensus sequence from the fastq files using MIA v1.0 (https://github.com/mpieva/mapping-iterative-assembler).

In addition to the 777 mitogenomes assembled here, we also retrieved 327 published mitogenome sequences, including 173 ancient whole mitogenome sequences and 154 modern samples. Consequently, the mitogenome dataset consists of 1104 sequences, including 989 domestic sheep, 43 Asiatic mouflon, 9 urial, 62 potential hybridized samples (*O. gmelini*/*aries*) and 1 outgroup (*O. canadensis*). All sequences were aligned using MAFFT v7.515 [24] and trimmed with trimAl v1.4 [25].

### Y-chromosomal SNPs

To obtain single-copy Y-chromosomal SNPs, raw SNPs in the 788 samples were processed as follows. Firstly, we identified ram samples from the whole samples using PLINK v1.90b6.26 [26] with the “check-sex” option and kept only these ram samples in the following analysis (see Additional file 1: Table S4). We then excluded SNPs in the regions covering the pseudo-autosomal regions (PARs) and the highly repetitive sequences (around 1.06 Mb) [14]. Afterwards, we filtered SNPs with the mean sequencing depth ranging from 0.5 × to 2 × of the expected depth (half of the genome-wide depth) [14]. Further, we removed SNPs that were heterozygous in at least one of the male samples. Finally, we retained 4096 Y-chromosome SNPs for the haplotype and phylogenetic analysis.

### Population structure analysis

To reveal the genetic differentiation within the Asiatic mouflon in Iran, we examined the population genetic structure for the total 788 mouflon, urial, argali and domestic samples using a set of autosomal SNPs filtered as follows. First, we filtered a total of 105,817,835 high-quality autosomal SNPs with missing rates < 0.1 and minor allele frequencies > 0.05 (MAF > 0.05) using PLINK v1.90b6.26 [26]. Next, 24,505,236 filtered SNPs were subjected to linkage disequilibrium-based pruning using PLINK v1.90b6.26 [26] with the parameters ‘–indep-pairwise 50 10 0.1’. This resulted in a final set of 849,728 SNPs retained for subsequent population genetic structure analysis.

We computed the identity by state among samples using PLINK v1.90b6.26 [26] with option “distance square 1-ibs”. We then constructed a neighbor-joining (NJ) tree using the “bionj” function from the R package *ape* v5.6.2 [27], and visualized it with the R package *ggtree* v3.6.2 [28]. Principal component analysis (PCA) was performed using the “-pca” option in PLINK v1.90b6.26 [26] with the default setting. Furthermore, model-based clustering was performed using sNMF v1.2 [29] with clusters *K* from 2 to 10.

The *F*_ST_ and nucleotide diversity (π) of autosomes were calculated using vcftools v0.1.17 [30] with 2Mbp windows and 1Mbp steps, and *P* value was obtained via 1000 permutations. *F*_ST_ values of mitochondria and Y chromosome were calculated using the R package *hierfstat* v0.5.11 [31] with 1000 permutations. The haplotype diversity of mitochondria and Y chromosomes were computed using pegas v1.1 [32] with 1000 permutations.

### Phylogenetic analysis

Phylogenetic analysis based on mitogenome and Y-chromosomal SNPs were performed using the maximum likelihood (ML) algorithm in iqtree2 v2.0.7 [33]. Mitogenome sequences were aligned using MAFFT v7.515 [24] with the default setting, and multiple sequence alignment (MSA) was trimmed to remove ambiguously aligned regions using trimAl v1.4 [25] with parameters “-automated1”. We further removed all gaps sites and ambiguous nucleotide bases, retaining only parsimony informative sites using *ClipKIT* [34]. We tested substitution models for DNA sequence using the option “-m” test in iqtree2 v2.0.7 [33] and identified the generalized time reversible model (GTR + G) [35] as the best fitting substitution model for mitogenome. We constructed a maximum likelihood (ML) tree with the site model GTR + G with 1000 bootstrap replicates using iqtree2 v2.0.7 [33]. The resulting tree was rooted with bighorn as the outgroup and visualized using FigTree v1.4.4 (http://tree.bio.ed.ac.uk/software/figtree/). Mitogenome haplotypes were inferred with the function “haplotype” in the R package *pegas* v1.1 [32]. We also constructed the parsimony mitogenome haplotype network using POPART [36]. We transformed the genotypes of Y-chromosomal 4096 SNPs into the fasta format using vcf2phylip.py [37]. Phylogenetic trees were built and visualized using the same pipeline, programs, and substitution model as described above.

### Demographic and divergence time inference

We inferred the dynamic changes in historical and recent effective population size (*N*_e_) using SMC++v1.15.2 [38]. We selected 6–10 high-depth individuals in each of the six geographically differentiated genetic groups of domestic sheep (Central-and-East Asian, South-and-Southeast Asian, the Middle Eastern, African, European, and American populations) [18] and two Iranian Asiatic mouflon subpopulations. We estimated the *N*_e_ during the past 1000 to 100,000 years for each group using SMC++v1.15.2 [38] under the default setting. Specifically, low-complexity regions of the reference genome were masked using snpable (http://lh3lh3.users.sourceforge.net/snpable.shtml, last accessed October 20, 2022) with in-house scripts. We set the mutation rate to be 1.00 × 10^–8^ per nucleotide site per generation [39] and generation times to 3 and 4 years, respectively [9, 40–42]. To infer the divergence times between *O. gmelini*_1 and *O. gmelini*_2, as well as between the Iranian Asian mouflon and domestic sheep, we employed the split option of SMC++ with the same parameter setting as above. We further estimate the *N*_e_ over the past 1000 years using GONe [43], with a parameter setting of 300 generations and a bin interval of 5 generations. To further investigate divergence and admixture events among Asiatic mouflon subspecies, we analysed whole-genome data from Urial sheep (*O. vignei*), Cyprus mouflon (*O. g. opion*), Anatolian mouflon (*O. g. anatolica*), and two additional *O. gmelini* lineages (*O. gmelini*_1 and *O. gmelini*_2) identified in this study, using bighorn sheep (*O. canadensis*) as an outgroup. Admixture graphs were inferred with OrientAGraph v1.0 [44] using parameters -mlno 1,2 -allmigs -bootstrap -k 5000. Models including one to five migration events were evaluated, and the topology with the highest likelihood was retained.

### Introgression analysis

We conducted introgression analysis to clarify the genetic contributions of two Iranian Asiatic mouflon subpopulations to South-and-Southeast Asian (SSA) sheep. We tested introgression using a combination method of *D* and *f*_dM_ statistics based on ABBA-BABA model. In the ABBA-BABA model, ABBAs refers to P2 and P3 sharing the derive allele B, and BABAs refers to P1 and P3 sharing the derive allele B. Under the introgression hypothesis between P2 and P3, a significant excess of ABBAs over BABAs is observed [45, 46]. Specifically, we calculated *D* statistics and *f*_4_-ratio [45] with a four taxon model (((P1, P2), P3), P4) using Dsuite v0.5r50 under the following format Eqs. (1) and (2) [46]:

1 $$D=\frac{{\sum }_{i=1}^{n}({\widehat{p}}_{i2}-{\widehat{p}}_{i1})({\widehat{p}}_{i3}-{\widehat{p}}_{iO})}{{\sum }_{i=1}^{n}({\widehat{p}}_{i2}+{\widehat{p}}_{i1}-2{\widehat{p}}_{i2}{\widehat{p}}_{i1})({\widehat{p}}_{i3}+{\widehat{p}}_{iO}-2{\widehat{p}}_{i3}{\widehat{p}}_{iO})},.$$

2 $${f}_{4}-\text{ratio}=\frac{{\sum }_{i=1}^{n}\left({\widehat{p}}_{i3a}-{\widehat{p}}_{iO}\right)\left({\widehat{p}}_{i2}-{\widehat{p}}_{i1}\right)}{{\sum }_{i=1}^{n}\left({\widehat{p}}_{i3a}-{\widehat{p}}_{iO}\right)\left({\widehat{p}}_{i3b}-{\widehat{p}}_{i1}\right)}.$$

where ${\widehat{p}}_{i1}$, ${\widehat{p}}_{i2}$, ${\widehat{p}}_{i3}$ and ${\widehat{p}}_{iO}$ are the derived allele of frequency at site *i* in population P1, P2, P3, and outgroup, respectively, and ${\widehat{p}}_{i3a}$ and ${\widehat{p}}_{i3b}$ are the derived allele of frequency at site *i* in two subpopulations P3a and P3b generated by randomly sampling from P3.

In this analysis, Menz sheep (MEN) was selected as the reference population (P1), while SSA populations were the target population (P2), *O. gemlini*_1 or *O. gemlini*_2 was the donor (P3), and bighorn sheep *O. canadensis* was the outgroup (P4). Evidence of gene flow between P3 and P2 was considered when *D* > 0, *f*_4_-ratio > 0, and *P*-value < 0.05, as determined by a standard block-jackknife procedure.

We further implemented sliding window statistics analysis (*f*_dM_) using the Dsuite v0.5r50 [46] to quantify introgressions and calculated *f*_dM_ values following format Eqs. (3) and (4):

3 $${f}_{dM}=\frac{\text{S}({P}_{1},{P}_{2},{P}_{3},\text{O})}{\text{S}({P}_{1},{P}_{d},{P}_{d},\text{O})},$$

4 $$\text{S}\left({P}_{1},{P}_{2},{P}_{3},\text{O}\right)={\sum }_{i=1}^{n}(1-{\widehat{p}}_{i1}){\widehat{p}}_{i2}{\widehat{p}}_{i3}(1-{\widehat{p}}_{iO})-{\sum }_{i=1}^{n}{\widehat{p}}_{i1}\left(1-{\widehat{p}}_{i2}\right){\widehat{p}}_{i3}\left(1-{\widehat{p}}_{iO}\right).$$

In here, we considered that derived alleles in P2 have higher or equal frequency than in P1 [46]. We calculated *f*_dM_ values using a sliding window of 50 SNPs with a step of 25 SNPs across the genomes with the defined trios (MEN, SSA, *O. gmelini*_2), where SSA represents the four South-and-Southeast Asian sheep populations suggested to be the introgressed populations by the *D*-statistics and *f*_4_-ratio tests. We calculated the Z-transformed *f*_dm_ values, and the windows with *P* < 0.05 and *D* > 0 were defined as the potential introgressed genomic regions. We evaluated the statistical significance using one-tailed *Z*-test in R package BSDA v1.2.1 [47].

We used *D*_FOIL_ v2017-011–25 [48] to infer the direction of gene flow between *O. gmelini*_2 and the four SSA introgression breeds. Based on the *D*_FOIL_ method, we designated the four SSA introgression breeds as P1, MEN as P2, and *O. gmelini_2* as P3, with *O. gmelina_*1 as P4 and bighorn as the outgroup. We used mvftools v0.6.2.1 [49] to count the ABBA-BABA pattern across the five populations. The dfoil.py and dfoil_analyze modules in *D*_FOIL_ were used to calculate the *D*-statistics and summarize the results.

### Selective sweep tests

To assess the potential roles of genomic introgression from Iranian Asiatic mouflon in Southern and Southeastern Asian (SSA) sheep populations, we conducted a Population Branch Statistics (*PBS*) analysis [50] to identify selective signatures in four introgressed populations (i.e., Garut (GUR), Nellore (NES), Bangladeshi (BGE), and Sumatra (SUM)). The *PBS* is calculated following format Eqs. (5) and (6):

5 $$PBS=\frac{{T}^{SM}+{T}^{SG}-{T}^{MG}}{2},$$

6 $$T=-\text{log}\left(1-{F}_{ST}\right).$$

Here, ${T}^{SM}$, ${T}^{SG}$, and ${T}^{MG}$ are the population divergence time *T* between four introgressed populations and MEN (${T}^{SM}$), four introgressed populations and *O. gmelini*_2 (${T}^{SG}$), and MEN and *O. gmelini*_2 (${T}^{MG}$). The *PBS* value shows the degree of allele frequency change at loci since divergence from the other two populations [50].

In our study, we pooled these four populations (GUR, BGE, SUM, and NES) as an introgressed group, and used the MEN and *O. gmelini*_2 as the reference populations. We calculated the *PBS* values using PBScan v2020.03.16 with parameters “-win 50 -step 25” described in Hämälä and Savolainen [51] and estimated *P* values by 2000 permutation cycles for Monte Carlo testing and detected selective regions with top 1% *PBS* values. Subsequently, we identified the overlaps between the selective and the introgressed regions using BEDtools v2.30.0 [52] and annotated functional genes in the overlapping genomic regions.

### Gene annotation and function analyses

We annotated genes in the introgressed regions using the sheep reference assembly *Oar_rambouillet_v1.0* by BEDtools v2.30.0 [52]. QTLs associated with these regions were identified using the sheepQTLdb (https://www.animalgenome.org/cgi-bin/QTLdb/OA/index) [53]. Linkage disequilibrium (LD) blocks were detected using LDBlockShow v1.40 [54] and LD decay was calculated with PopLDdecay v3.42 [55]. The *d*_xy_ and *F*_ST_ values for the introgressed regions were calculated using Pixy 1.2.7.beta1 [56] with a 20 kb sliding window and 2 kb steps. Haplotype heatmaps surrounding the introgression regions were generated using R package *ComplexHeatmap* v2.14.0 [57].

## Results

### Genetic variants, mitogenome assembly, and Y-chromosome specific SNPs

A total of 105,817,835 SNPs in the 788 wild and domestic sheep samples were obtained for downstream analyses. We obtained 777 samples with an average depth of 933.23 × (101.92 × − 8324.72 ×) across the entire mitogenome (see Additional file 1: Table S2) and assembled the mitogenome of 777 samples by aligning all mitochondrial reads to the reference mitochondrial genome NC_001941.1. After quality control for the SNP identification on the Y-chromosome, we identified 4096 Y-specific SNPs in a cohort of 239 rams with an average read depth of 8.26 × (~ 4.25 × – 16.99 ×) (see Additional file 1: Table S4). Totally, 1104 whole mitogenomes (including 105 from Asiatic mouflon), 788 whole-genome sequences (40 from Asiatic mouflon), 239 Y chromosomes (21 from Asiatic mouflon), and 337 *Cyt*-b sequences (244 from Asiatic mouflon) were used to conduct a comprehensive population genomics analysis of Asiatic mouflon (see Additional file 1: Table S5).

### Population genetic structure of Iranian Asiatic mouflon

**Fig. 1:**
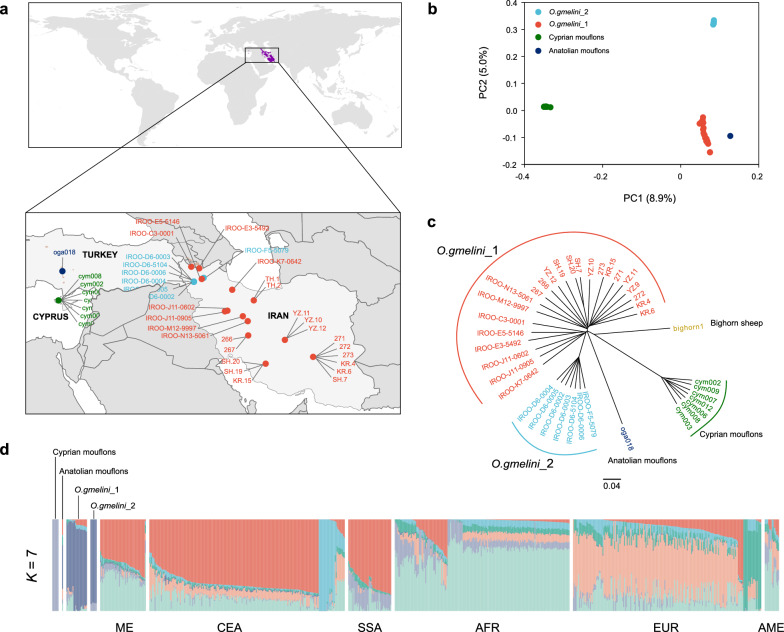
Population genetics structure of Asiatic mouflon in Iran (*O. gmelini*). **a** Geographic distribution of Asiatic mouflon used in this study. Purple, light blue, green and dark blue dots indicate the location of *O. gmelini*_1, *O. gmelini_2*, Cyprian mouflon and Anatolian mouflon; **b** Principal components 1 and 2 for Asiatic mouflon; **c** Neighbor-joining (NJ) trees of Asiatic mouflon based on whole-genome SNPs, using bighorn sheep as outgroup; **d** Population genetic structure of the Asiatic mouflon and domestic sheep inferred from the program the sNMF v1.2 (*K* = 7) using whole-genome SNPs

Iranian Asiatic mouflon presented clear genetic divergence with Cyprian mouflon, Anatolian mouflon, urial and bighorn sheep (see Additional file 2: Figures S1 to S2). Autosomal SNP analysis (e.g., PCA, NJ-tree and admixture) revealed two distinct subpopulations among the 32 Iranian Asiatic mouflons (Fig. 1b–d), designated as *O. gmelini*_1 and *O. gmelini*_2. However, TH.1 from Iranian Asiatic mouflon clustered with urial, consistent with the reported hybridization between Asiatic mouflon and urial in Iran [58, 59], and TH.2 also showed close affinity to urial (see Additional file 2: Figures S3–S4). Therefore, TH.1 and TH.2 as potential hybridized samples were removed in the following analysis. *O. gmelini*_1 comprised 23 samples across eastern and southern Iran, while *O. gmelini*_2 consisted of only 7 samples limited in northwestern Iran (Fig. 1a–c). Low kinship values within two subpopulations eliminate inbreeding caused by sampling (see Additional file 1: Table S6). We also observed significant genetic divergence (Table 1) and different genetic diversity patterns among both subpopulations (Table 2). Autosomal SNP analysis, including phylogenetic tree construction and admixture analysis, revealed six genetic clusters of domestic sheep, consistent with their geographic distribution (Fig. 1d and see Additional file 2: Figures S1, S5 and S6).

**Table 1:** The *F*_ST_ of autosome, mitogenome and Y chromosome of *O. gmelini*_1, *O. gmelini*_2 and *O. aries*

Genome_Type	Pop1	Pop2	*F*_ST_	*P*-value
Autosome	*O. aries*	*O. gmelini*_1	0.1186	*P* < 0.05
	*O. aries*	*O. gmelini*_2	0.2167	*P* < 0.05
	*O. gmelini*_1	*O. gmelini*_2	0.1640	*P* < 0.05
Mitogenome	*O. aries*	*O. gmelini*_1	0.5491	*P* < 0.001
	*O. aries*	*O. gmelini*_2	0.7211	*P* < 0.001
	*O. gmelini*_1	*O. gmelini*_2	0.3213	*P* < 0.005
Y-chromosome	*O. aries*	*O. gmelini*_1	0.7354	*P* < 0.001
	*O. aries*	*O. gmelini*_2	0.5494	*P* < 0.001
	*O. gmelini*_1	*O. gmelini*_2	0.2140	*P* < 0.001

**Table 2:** The nucleotide diversity/haplotype diversity of autosome, mitogenome and Y chromosome of *O. gmelini*_1, *O. gmelini*_2 and *O. aries*

Genome_Type	Pop	Mean	Sd	Parameter
Autosome	*O. aries*	0.00018	5.90232E-05	Nucleotide diversity
	*O. gmelini_*1	0.00017	5.82569E-05	
	*O. gmelini_*2	0.00011	4.53947E−05	
Mitogenome	*O. aries*	0.99812	1.29451E−07	Haplotype diversity
	*O. gmelini_*1	0.98913	1.43693E−04	
	*O. gmelini_*2	0.47619	2.71712E−02	
Y-chromosome	*O. aries*	0.80593	4.08609E−04	Haplotype diversity
	*O. gmelini_*1	0.98901	5.90413E−04	
	*O. gmelini_*2	0.28571	4.01023E−02	

### Phylogenetic and haplotype analysis based on uniparental variants

**Fig. 2:**
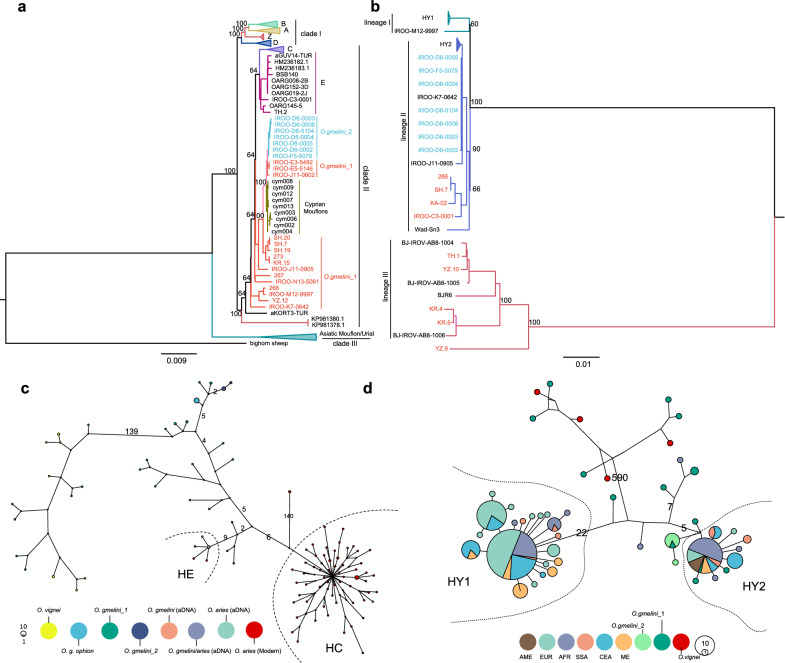
Phylogeny of Asiatic mouflon, urial, and domestic sheep inferred from mitogenomes and Y-chromosomal genetic variants. **a** Phylogeny of Asiatic mouflon, urial, and domestic sheep inferred from 767 mitogenomes; **b** Phylogeny of Asiatic mouflon, urial, and domestic sheep inferred from 239 Y-chromosomal SNPs; **c** Network of mitogenome haplotypes; **d** Network of Y-chromosome haplotypes. Bootstrapping values above 50% were labeled on the phylogenetic tree. Bighorn sheep were used as the outgroup in phylogenetic analyses based on mitogenome and Y-chromosome genetic variants

We identified 989 mitochondrial haplotypes from the 1104 mitogenomes of wild and domestic sheep (Fig. 2a and see Additional file 1: Table S2). The phylogenetic tree inferred from the mitochondrial haplotypes revealed three monophyletic clades (clades I, II, and III) (Fig. 2a). Clade I comprised of haplogroups A, B, Z, and D from domestic sheep, with Z found exclusively in Neolithic samples [15]. Clade II included haplogroups C and E from domestic sheep, along with some Asiatic mouflon samples from Iran, Turkey and Cyprus, while the remaining Iranian Asiatic mouflon samples clustered with the urial in clade III (Fig. 2a).

The phylogenetic tree inferred from Y-chromosomal SNPs revealed three lineages (Fig. 2b) and (see Additional file 1: Table S7). Lineage I comprised of haplogroups HY1 from domestic sheep and 1 sample from *O.gmelini*_1 (IROO-M12-9997). Lineage II included Haplogroups HY2 from domestic sheep and some Iranian Asiatic mouflon samples, and the remaining Iranian Asiatic mouflon samples clustered with urial in Lineage III. Notably, lineage II demonstrated a close affinity between HY2 and Iranian Asiatic mouflon (Fig. 2b). However, phylogenetic and network analysis revealed no shared mitochondrial haplotypes and only one shared Y-haplotype between domestic sheep and Iranian Asiatic mouflon in HY2 (Fig. 2c–d). The results and significant genetic divergence (*O. gmelini*_1: *F*_ST_ = 0.11, *P* < 0.05; *O. gmelini*_2: *F*_ST_ = 0.21, *P* < 0.05) between Iranian Asiatic mouflon and domestic sheep observed by autosome SNPs (Table 1), suggest that Iranian Asiatic mouflon may not be the direct ancestor of domestic sheep. Instead, the relatively close phylogenetic relationships between the Asiatic mouflon in Iran and domestic sheep suggested by haplogroups C, E, and Y-chromosomal haplogroupY2 imply possible gene flows between these groups.

### Effective population size and divergence time

**Fig. 3:**
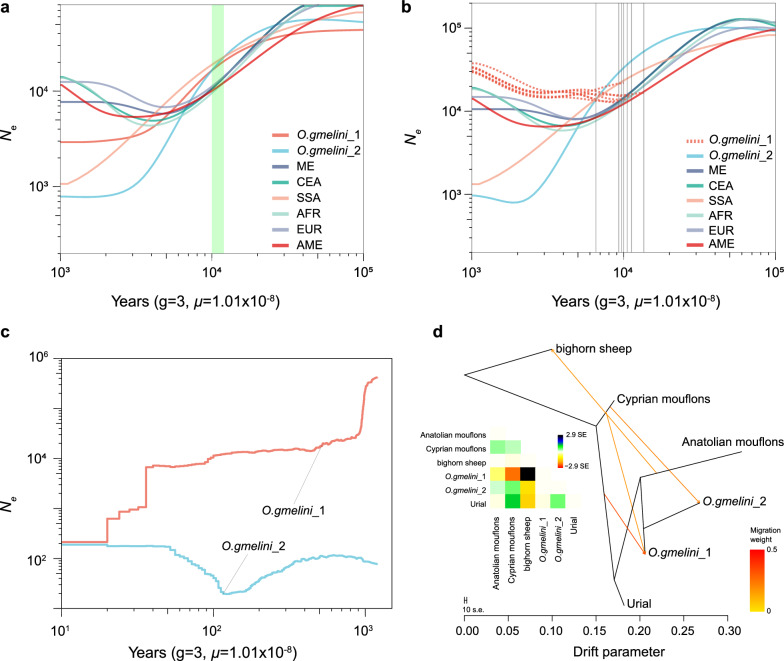
Demographic history of Iranian Asiatic mouflon and domestic sheep. **a** Effective population sizes (*N*_e_) across six domestic sheep populations and the two subpopulations of Asiatic mouflon in Iran inferred from SMC++; **b** Inference of split times between *O. gmelini*_1 and the other wild and domestic sheep populations. The vertical lines represent the split time between each pair of populations.; **c** Effective population size inference of *O. gmelini*_1 and *O. gmelini*_2 using GONe; **d** Maximum Likelihood Network Orientation reconstruction for the relationship between *O. gmelini*_1 and *O. gmelini*_2 using OrientAGraph

Inference of demographic history showed the Asiatic mouflon and domestic sheep shared a similar historical pattern 10,000 years ago (Fig. 3a, b). SMC++ analysis revealed a continual decline in *N*_e_ in domestic sheep and Asiatic mouflon during ~ 8000–10,000 years ago, while the two Iranian Asiatic mouflon populations exhibited slower declines than domestic sheep (Fig. 3b). A sharp decline in *N*_e_ was detected ~ 1000–5000 years ago in the *O. gmelini*_2, demonstrating a pattern different from that observed in *O. gmelini*_1 (Fig. 3a). We observed similar divergence times between *O. gmelini*_1 and 6 geographically differentiated genetic groups of domestic sheep during ~ 9000-15,000 years ago (Fig. 3b and Table 3). Additionally, we found the splitting time between the two Iranian Asiatic mouflon populations around ~ 6567-8200 years ago (Fig. 3b and Table 3), indicating a recent genetic divergence of Iranian Asiatic mouflon. The GONe analysis supported divergence between *O. gmelini*_1 and *O. gmelini*_2 over the past 1000 years and revealed more strength bottleneck in *O. gmelini*_2 compared to *O. gmelini*_1 during this period (Fig. 3c). OrientAGraph analysis revealed no significant admixture between *O. gmelini*_1 and *O. gmelini*_2 (see Additional file 2: Figure S7). The optimal model, incorporating four migration events, displayed the highest likelihood score (See Additional file 1: Table S8) and supported gene flow between Cyprus mouflon (*O. g. ophion*) and both *O. gmelini*_1 and *O. gmelini*_2 (Fig. 3d).

**Table 3:** Split times estimated by SMC++

Split pair	Split time (year) (generation = 3 years)	Split time (year) (generation = 4 years)
Split_time_AFR vs*O. gmelini*_*_*_1_	11,254.1	15,005.5
Split_time_AME vs*O. gmelini*_*_*_1_	13,581.1	18,108.2
Split_time_CEA vs*O. gmelini*_*_*_1_	9285.16	12,380.2
Split_time_EUR vs*O. gmelini*_*_*_1_	10,534.9	14,046.5
Split_time_ME vs*O. gmelini*_*_*_1_	9695.75	12,927.7
Split_time_*O. gmelini*_*_*_2 vs*O. gmelini*_*_*_1_	6567.53	8209.4
Split_time_SSA vs*O. gmelini*_*_*_1_	9991.41	13,321.9

### Phylogeography of Asiatic mouflon

**Fig. 4:**
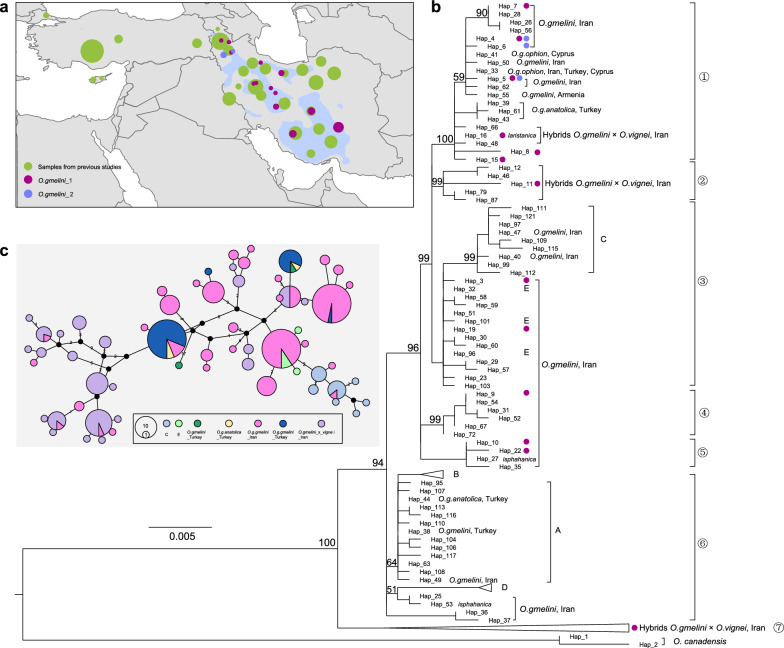
The phylogenetic relationship among Asiatic mouflon subspecies and domestic sheep. **a** Geographical distribution of Asiatic mouflon and samples information of 32 Asiatic mouflon in Iran and 301 domestic sheep samples; **b** Phylogenetic tree inferred based on complete *Cyt-b* sequences using the maximum likelihood algorithms. Bootstrap values above 50% were labeled on the phylogenetic tree. **c** Network of Asiatic mouflon in Iran and Turkey and domestic sheep lineage of C and E built from complete *Cyt-b* sequences

We built a *Cyt-b* dataset comprising 247 Asiatic mouflon and 8 European mouflon. The samples of Asiatic mouflon covered the current distribution of all the subspecies (Fig. 4a and see Additional file 1: Table S3). We performed phylogenetic analysis of *Cyt-b* sequences of Asiatic mouflon and domestic sheep, using argali as the outgroup. In the phylogenetic tree built from *Cyt-b*, Asiatic mouflon was grouped into 7 major clades supported by high bootstrap values (94–100) (Fig. 4b). The topology revealed 5 domestic sheep mitogenome clades (A, B, C, D, and E). Notably, haplogroups C + E and Asiatic mouflon samples from Iran clustered into a monophyletic clade (clade 3) with high frequencies of shared haplotypes between them (Fig. 4c and see Additional file 1: Table S9). The results were consistent with the mitogenome analyses and suggest that partial haplotypes C + E in domestic sheep may have originated from Iranian Asiatic mouflon by introgression. Haplogroups A + B + D in domestic sheep and Asiatic mouflon samples from Iran and Turkey fell within clade 6, however no common haplotypes were identified between domestic and wild sheep (Fig. 4c and see Additional file 1: Table S9). Furthermore, the majority hybrid samples formed a monophyletic clade (clade 7), while approximately 10% of the hybrid samples clustered with subspecies, such as *O.g.ophion* and *O.g.anatolica* (Clade 1 and 2) (Fig. 4b and see Additional file 1: Table S9).

### Genetic introgression

**Fig. 5:**
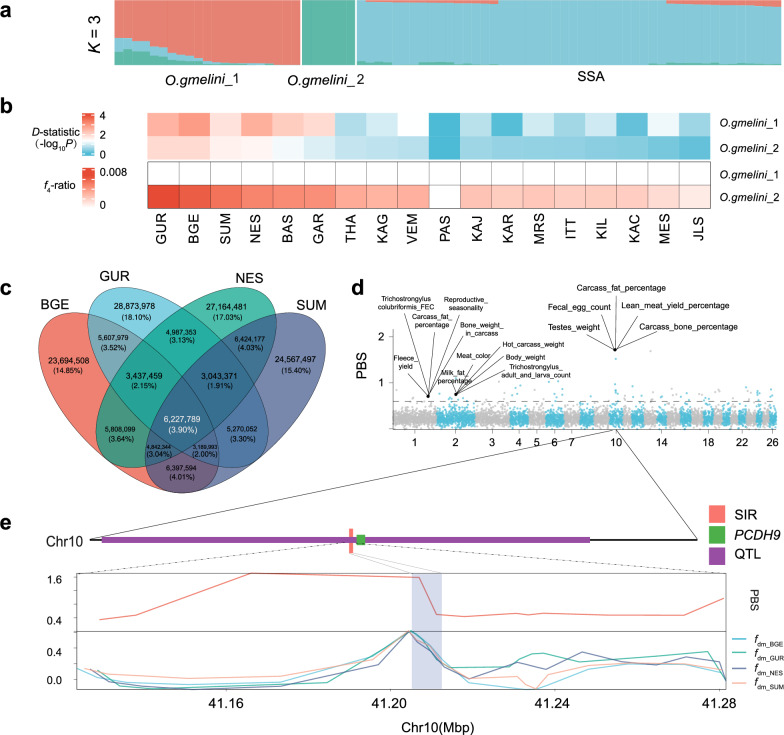
Genetic introgression of Asiatic mouflon in SSA domestic populations. **a** Admixture proportions of Asiatic mouflon in Iran and SSA domestic populations inferred from the program the sNMF v1.2 (*K* = 3) using whole-genome SNPs. **b** Heatmap of *D*-statistics and *f*_4_-ratio calculated by Dsuite v0.5 applied to data including *O. gmelini*_1, *O. gmelini*_2, and SSA domestic populations; **c** Venn diagram for the shared introgression regions among four SSA domestic populations; **d** Manhattan plot showing signals of selection in the four introgressed SSA populations (BGE/NES/GUR/SUM) detected by the *PBS* with the dashed line marking 99.9% threshold. The sheep QTLs overlapped with the top 1% PBS regions are shown, **e** *PBS* and *f*_dm_ values around the significant introgressed genomic region on Chr.10. The selected introgression region (SIR), QTL, and adjacent gene *PCDH9* were labeled with coral, purple, and green bars, respectively

The SMC++ analysis revealed a similar demographic history between *O. gmelini*_2, and SSA (Fig. 3a, b), suggesting potential gene flow between these populations. To further investigate the impact of introgression among Iranian Asiatic mouflon and SSA, we conducted introgression tests between these groups. We first conducted an admixture analysis between Iranian Asiatic mouflon and the SSA populations using sNMF [29]. We identified three genetic clusters with the smallest cross validation error (*K* = 3) (*O. gmelini*_1, *O. gmelini*_2, and SSA) and detected identical genetic components between Iranian Asiatic mouflon and some domestic populations (Fig. 5a and see Additional file 2: Figure S8). Further, we calculated Patterson’s *D*-statistic and *f*_4_-ratio with the ABBA-BABA model to assess introgression between *O. gmelini*_1 or *O. gmelini*_2 (P3) and SSA populations (P2) based on the criteria: *D* > 0, *P*-value < 0.05, and *f*_4_-ratio > 0. Genetic introgression from *O. gmelini*_2 was detected in only four sheep populations (GUR, NES, BGE, and SUM) (Fig. 5b and see Additional file 1: Table S10), all of which are located in regions adjacent to the Indian Ocean. *D*_FOIL_ tests support introgression from *O. gmelini*_2 into the four SSA breeds [DFO = 0.433 (*P* = 0.042), DIL = 0.005 (*P* = 0.083), DFI = 0.712 (*P* = 0.034), DOL = 0.378 (*P* = 0.048)].

We identified introgressed regions with top 5% *f*_dm_ and *D* > 0, revealing a common introgression region across the four populations spanning 6,227,789 bp (Fig. 5c and see Additional file 1: Tables S11 to S12). We then calculated the *PBS* value of the four populations to identify regions under selection, using MEN sheep and *O. gmelini*_1 as the reference populations. Regions within the top 1% of *PBS* values were under selection (Fig. 5d and see Additional file 1: Table S13). We identified a 19,614 bp overlapping region as the selected introgressed region (see Additional file 1: Table S14). By intersecting the selected introgressed regions with sheep QTLs, we identified associations with traits such as carcass fat percentage, body weight, and carcass weight (see Additional file 1: Table S14 and Additional file 2: Figure S9).

## Discussion

Asiatic mouflon had been classified into five subspecies using phenotypic traits, such as coat color, horns, and geographic patterns [12]. However, the intraspecies taxonomy has long lacked genetic evidence and the genetic contribution of these populations to domestic sheep remains unclear. Here, we identified two genetic clusters of Iranian Asiatic mouflon based on WGS data: *O. gmelini*_2 limited in Kaboudan Island in Urmia Lake National Park, and *O. gmelini*_1 over a wide geographic area in Iran (Fig. 1). We estimated the divergence time of the two clusters to be approximately 6567–8200 years ago (Fig. 3b, Table 3), indicating a recent split. This finding is further corroborated by the phylogenies inferred from uniparental genetic markers (Fig. 2a, b).

All individuals of *O. gmelini*_2 collected from Kabudan Island in Urmia Lake National Park, except for 1 sample (IROO-F5-5079), inhabited the overlap geographic region with Armenian Mouflon (*O. g.gmelini*) [11]. In contrast, individuals of *O. gmelini*_1 were sampled from the distribution areas of several subspecies such as Armenian Mouflon (*O. g.gmelini*), Isfahan mouflon (*O.g.isphahanica*), and Laristan mouflon (*O.g.laristanica*) (Fig. 1a) [11]. *O. gmelini*_1 clustered with urial, with TH.1 and TH.2, exhibiting a continuous genetic affinity with urial (see Additional file 2: Figures S1 to S4). The results suggest potential admixture between both species. Compared to *O. gmelini*_1, *O. gmelini*_2, the lower genetic diversity (Table 2) and smaller *N*_e_ (Fig. 3) suggest a founder effect or bottleneck, consistent with the introduction history of Armenian mouflon to Kabudan Island in Urmia Lake in 1895 and 1906 [60, 61]. Therefore, our results suggest that *O. gmelini*_2 may represent Armenian Mouflon and exhibits significant genetic divergence from other subspecies. However, the lack of phenotypic data and the limited sampling constrains our ability to fully understand genetic differentiation within species. For instance, we cannot distinguish between genetic divergence resulting from founder effects or bottlenecks caused by isolation, long-term selection or genetic drift.

Furthermore, international collaborations should be strengthened to broaden the sampling of Asiatic mouflon across its natural distribution area and to collect phenotypic data, such as horns [61] and body size [12]. It’s necessary to carry out phylogenetic analyses for Asiatic mouflon samples to overcome the drawback of the absence of phenotypic data and clarify genetic divergence using multiple markers, for example whole-genome variations and uniparental markers. Also, Asiatic mouflon is under the status of “Near Threatened”, and their population size has declined in the past two decades under competition with domestic sheep, poaching, and habitat deterioration [11, 62]. Therefore, the population genetic structure analyses and delimitation of taxonomic classification based on WGS data are fundamental for conservation decisions and effective conservation management [63, 64]. For example, clarifying the sub-species delimitation, genomic diversity, and demographic histories to assess endangered status for scientific conservation decisions and investigating comprehensive genetic evidence to suggest conservation management units.

Genetic variants of uniparental inheritance offer insights into the domestication and evolutionary history of farm animals, such as pigs [65], sheep [6, 14, 15] and goats [66, 67]. Here, we identified 6 maternal haplogroups (A, B, C, D, E, and Z) in two monophyletic groups (A + B + D + Z and C + E), and two paternal clades (HY1 and HY2) (Fig. 2a, b). We found that the majority of the haplotypes from Iranian Asiatic mouflon clustered with mitochondrial haplogroups C + E (24/31) and with Y-chromosomal haplogroup HY2 (12/21) (see Additional file 1: Table S7). Those haplotypes exhibited greater divergence from mitochondrial haplogroups A + B and Y-chromosomal haplogroup HY1 (Fig. 2c, d). Uniparental phylogenetic patterns of Iranian Asiatic mouflon were discordant with those inferred by autosomal SNPs, as we did not observe two subpopulations corresponding to *O. gmelini*_1 and *O. gmelini*_2 using uniparental markers. This discordance patterns may be explained by recent hybridization events [68], but further validation with additional samples is required.

Additionally, *Cyt-b* sequences sampled across the entire geographic areas of Asiatic mouflon also revealed a close relationship between Iranian Asiatic mouflon and domestic sheep belonging to maternal haplogroups C + E (Fig. 4), consistent with results from the mitogenome. Considering an ancient mitogenome study that reported a 5% frequency of haplogroup E and the absence of haplogroups C in southwestern Anatolia approximately 7720 years ago [15], our findings suggest that haplogroups C may have originated from Iranian Asiatic mouflon. However, WGS analysis revealed intraspecies substructure in Iranian Asiatic mouflon, and genetic introgression from the *O. gmelini*_2 showed a larger impact on domestic sheep than *O. gmelini*_1 (Fig. 5). Similar results were observed from Alberto et al. that IROO individuals were split into subpopulations and had admixtures with some domestic sheep [41]. Given that *O. gmelini*_2 may have been introduced from Armenian mouflon to Kabudan Island in Urmia Lake in 1895 and 1906, our findings highlight the complexity of the relationship between Asiatic mouflon and domestic sheep. In summary, our results suggest that Iranian Asiatic mouflon may not be the direct ancestor of domestic sheep [5, 15]. However, they could have contributed to the gene pool of domestic sheep through multiple introgression events. A comprehensive understanding of the history of domestic sheep requires further validation through the integration of paleogenomic and modern genomic data from both domestic sheep and their wild relatives.

Our results revealed the genetic introgression from Iranian Asiatic mouflon into several South and Southeast Asian sheep populations, supporting previous findings [9, 19]. Specifically, we detected genetic introgression in four sheep populations: Garut, Bangladeshi, Nellore, and Sumatra, which were exclusively from *O. gmelini_*2 on Kabudan Island in Urmia Lake National Park (Fig. 5b). The introgression event aligns with the demographic history of *O. gmelini_*2 and South and Southeast Asian sheep inferred both here and in previous studies indicates that South and Southeast Asian sheep descended from Central and East Asian populations around 5850–6010 years ago with potential admixture from Middle Eastern sheep 3930–4180 years ago [18], and *O. gmelini_*2.

In the four sheep populations, we found that common introgression signals from *O. gmelini_*2 covered about 3.90% of the total introgressed regions. Compared to population-specific introgression, common introgression could be related to a specific trait or adaptation to similar environments, such as *EPAS1* for the adaptation to hypoxia [69]. We further integrated *PBS* analyses and found four positive selective regions in the common introgressed regions, which were related to carcass fat percentage, body weight, carcass weight (Fig. 5d). This might have contributed to the small body size phenotype or adaptation to hot and humid environments of Garole, Sumatra, and Bangladeshi sheep [70–72]. The results imply that the adaptive selected introgressed region may have been introgressed from *O. gmelini*_2, and then have undergone strong selection to facilitate their adaptation to the hot and humid environments near the Indian Ocean.

**Fig. 6:**
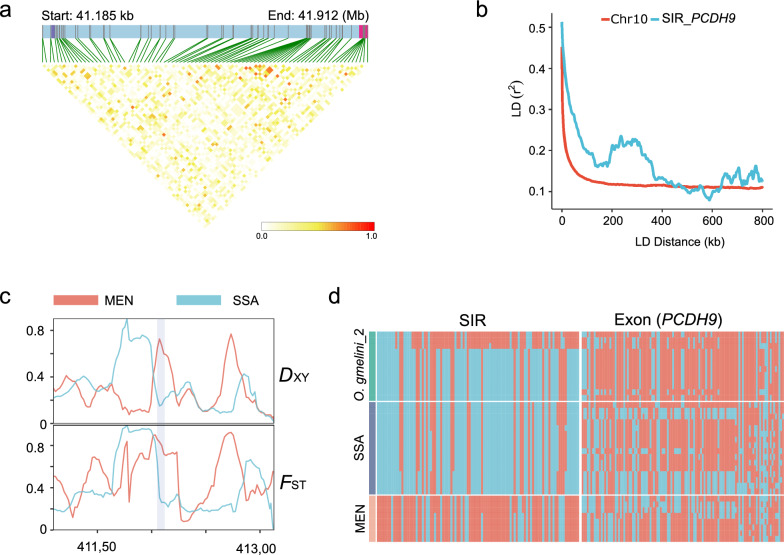
Candidate gene *PCDH9* associated with the significant introgression signal on Chr. 10. **a** Linkage disequilibrium (LD) blocks in the significant introgression region and gene *PCDH9* (chr10:41,104,271-43,073,231). The purple and pink bars represent the location of the significant introgression region and gene *PCDH9*; **b** Pattern of LD decay in the introgressed populations. The red line is calculated from the whole 10 chromosomes, the green line is calculated from the beginning of the significant selected introgression region (SIR) (chr10:41,104,271) to the next 800 kb; **c** *D*_xy_ and *F*_ST_ around the significant introgression region on Chr. 10 (chr10:41,104,271–41,313,804), calculated by pixy v1.2.7.beta1; **d** Haplotype pattern of the significant selected introgression region (SIR) on Chr. 10 (chr10:41,204,271–41,213,804) (left) and exons of *PCDH9* (right)

We identified a candidate gene *PCDH9* located approximately 679 kb downstream of a positive selective region within the common introgressed region (Fig. 5e). This finding was supported by linkage analysis (Fig. 6a, b) and validated by *d*_XY_ and *F*_ST_ of Menz sheep (MEN) and introgressed breeds (BGE, NES, GUR, and SUM) (Fig. 6c). An interesting finding is that similar haplotype patterns were observed in the region among introgressed breeds and *O.gmelini*_2, rather than in the exon of *PCDH9* (Fig. 6d), suggesting that the region may regulate the function of *PCDH9*. *PCDH9* had been reported to be associated with local adaptation (e.g., hot and arid environments) in sheep and goats [73, 74] and fat deposition [75]. Further analysis using Chip-seq and RNA-Seq data will be required to uncover the functional and regulatory mechanisms of *PCDH9* in adaptation to hot and humid environments in sheep.

## Conclusions

In summary, we conducted a comprehensive and integrated analysis of Asiatic mouflon and domestic sheep based on WGS, mitogenome and Y-chromosomal genetic variants. Our results suggest that the mitochondrial haplogroup C of domestic sheep may have originated from the Iranian Asiatic mouflon through introgression, further clarifying the role of the Iranian Asiatic mouflon in the domestication process. Additionally, our results identified the introgression signals from Iranian Asiatic mouflon in several domestic sheep populations in South and Southeast Asia, indicating that the introgression may have facilitated their adaptation to hot and humid environments near the Indian Ocean. Our findings provide new insights into the domestication and subsequent evolutionary history of sheep.

## Supplementary Information

Additional file1: Table S1. 788 samples information for autochromosome analysis. Table S2: 1104 samples information for mitochondria chromosome analysis. Table S3 337 samples information of *Cyt-b* sequence in this study. Table S4. 239 samples for Y chromosome analysis in this study. Table S5. Summary information of datasets for various analyses in this study. Table S6. Kinship of 32 *O. gmelini* samples. Table S7. Haplotype information of mitogenomes and Y chromosomes*.* Table S8. Likelihood estimates for different migration number calculated using OrientaGraph. Table S9. Haplotype information of *Cyt*-*b* sequences. Table S10. *D*-statistics from Iranian Asiatic mouflon to SSA populations. Table S11. Details for chromosome-level introgression across four breeds. Table S12. Common introgressed blocks from *O.gmelini*_2 into four SSA populations. Table S13. Population branch statisticof putative selective regions in the SSA population. Table S14. Overlaps regions among selected regions identified by *PBS*, introgression regions and QTLs regions. Table S15. Softwares used in this study.

Additional file 2: Figure S1. Title: Neighbor-joiningtree of wild and domestic sheep based on whole-genome SNPs. The tree showed the same two clusters of *O. gmelini *as Fig.1b and domestic sheep samples clustered based on their geographic distribution. Figure S2. PCA of wild and domestic sheep based on whole-genome SNPs. Figure S3. Neighbor-joiningtree of Asiatic mouflon based on whole-genome SNPs using bighorn sheep as the outgroup. Figure S4. PCA of Asiatic mouflon and urial based on whole-genome SNPs. Figure S5. Neighbor-joiningtree of the Asiatic mouflon and domestic sheep. Figure S6. Admixture of Asian mouflon and domestic sheep for *K*=2-9. Figure S7. Maximum Likelihood Network Orientation reconstruction for the relationship between *O. gmelini*_1 and *O. gmelini*_2 using OrientAGraph. a migration event = 1; b migration events = 2; c migration events = 3; d migration events = 5. Figure S8. Admixture of 18 SSA breeds with two Iranian Asiatic mouflon populations for *K*= 2-9. Figure S9. Word cloud summarizing QTL traits associated with the overlapping regions of *PBS* selection and introgression.

## Data Availability

The whole genome re-sequence data used for the study is publicly available under the sample accession numbers listed in Additional file 1: Table S1. All mitogenomes assembled in this study were deposited in NCBI’s SRA under the accession number OR160429-OR161034 and PQ247876-PQ248020. All software used in this study and links of those software were listed in Additional file 1: Table S15. All scripts used for this work were performed using open-source software tools and are available from the corresponding authors upon request.
