# Supplementary material for: Genomic analyses of Asiatic Mouflon in Iran provide insights into the domestication and evolution of sheep
Source: Genet Sel Evol. 2025 Jun 13;57:31. doi: 10.1186/s12711-025-00978-y (PMC12164106; doi:10.1186/s12711-025-00978-y)

**Figure S1**. Neighbor-joining (NJ) trees of wild and domestic sheep based on whole-genome SNPs. The tree showed the same two clusters of *O. gmelini* as Fig.1b and domestic sheep samples clustered based on their geographic distribution.


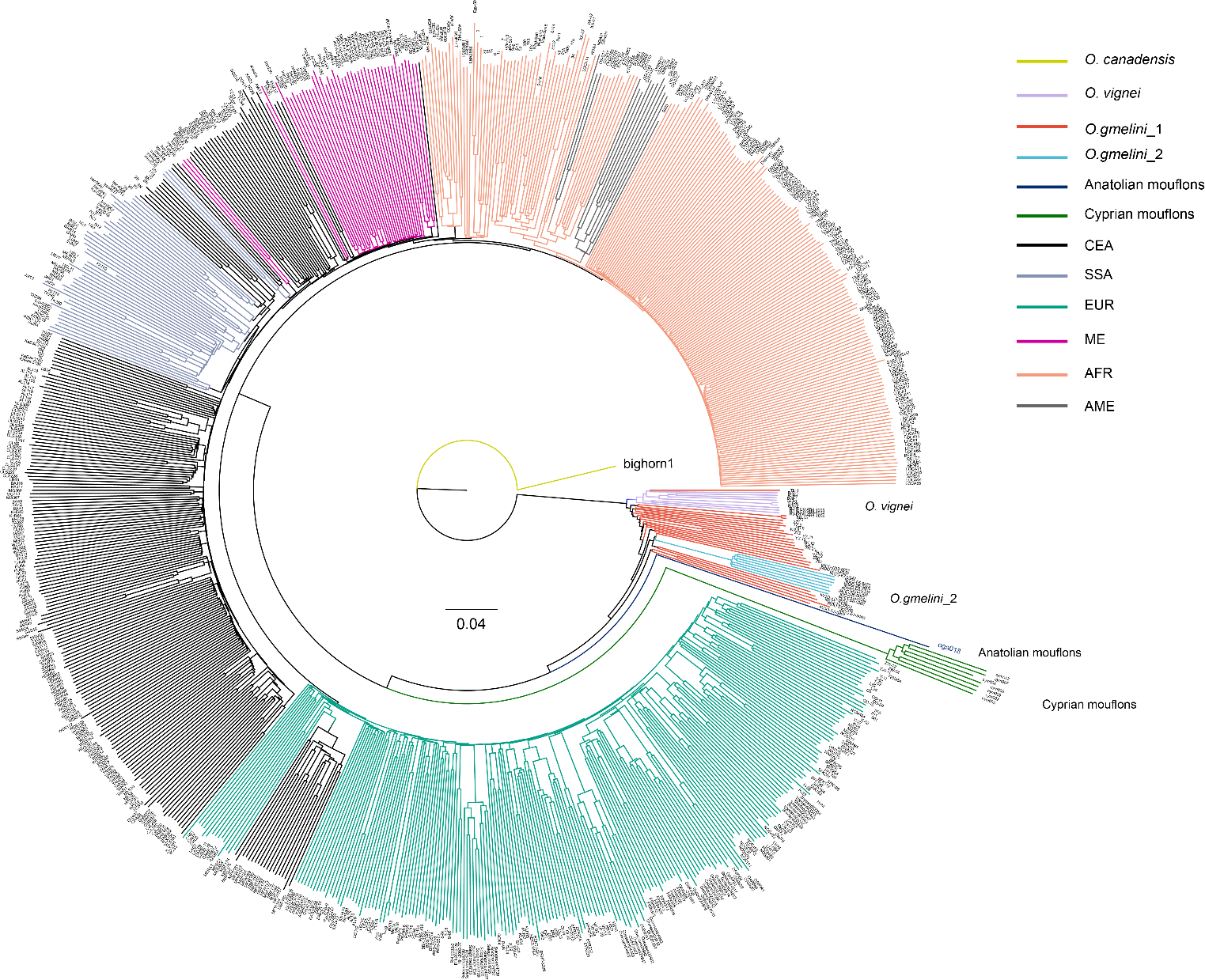


**Figure S2.** PCA of wild and domestic sheep based on whole-genome SNPs.


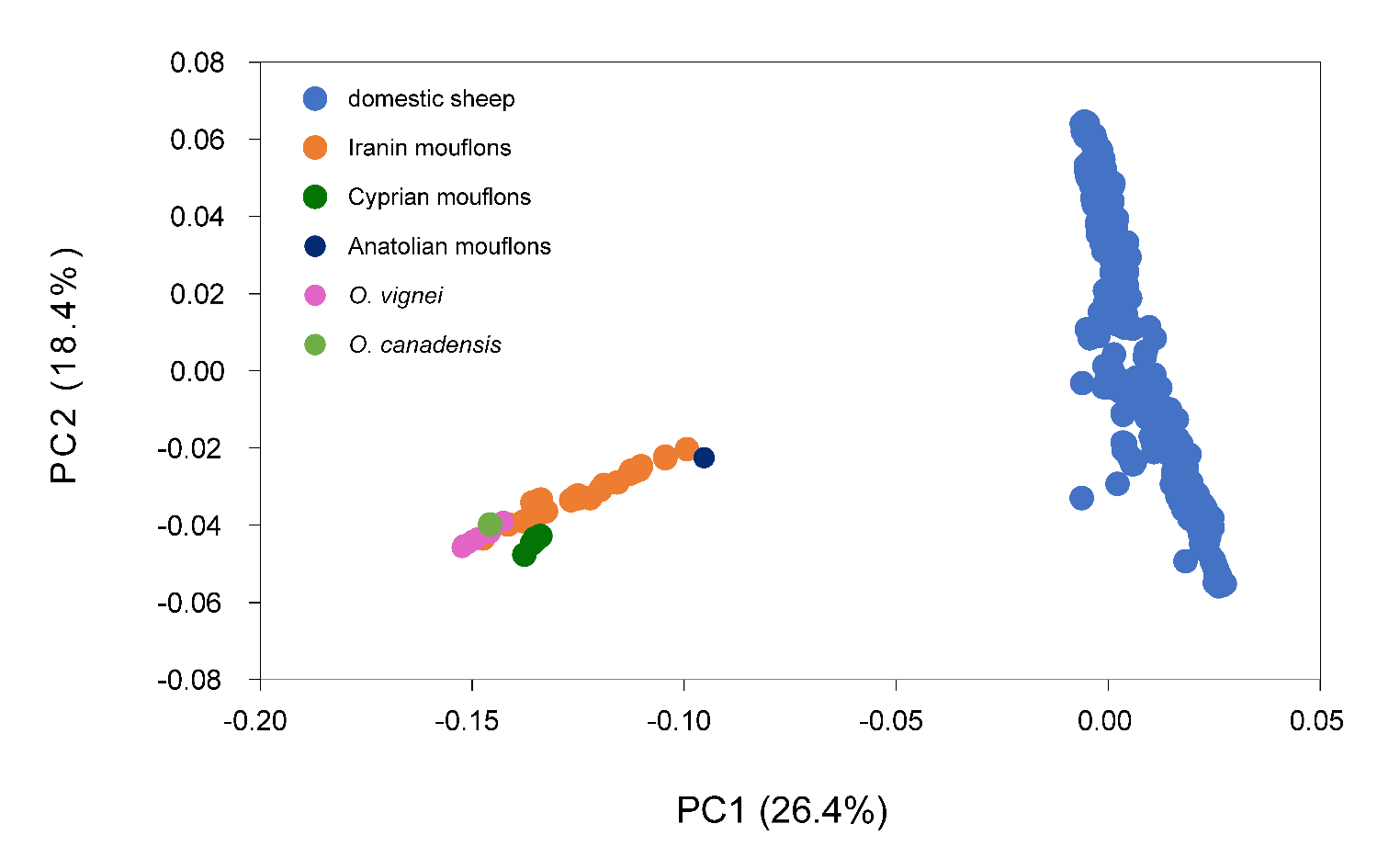


**Figure S3.** Neighbor-joining (NJ) trees of Asiatic mouflon based on whole-genome SNPs using bighorn sheep (*O. canadensis*) samples as the outgroup.


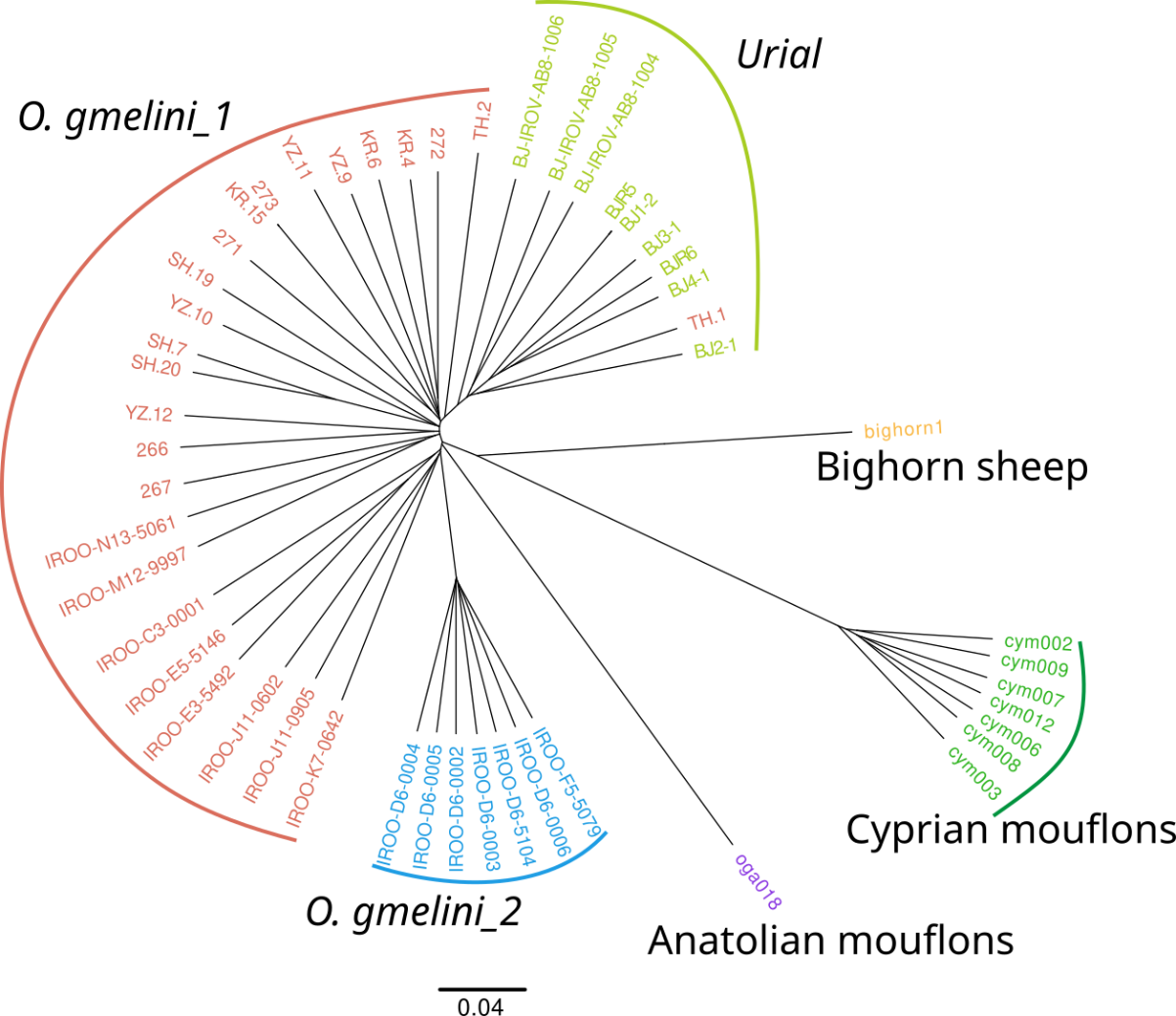


**Figure S4.** PCA of Asiatic mouflon and urial based on whole-genome SNPs.

**
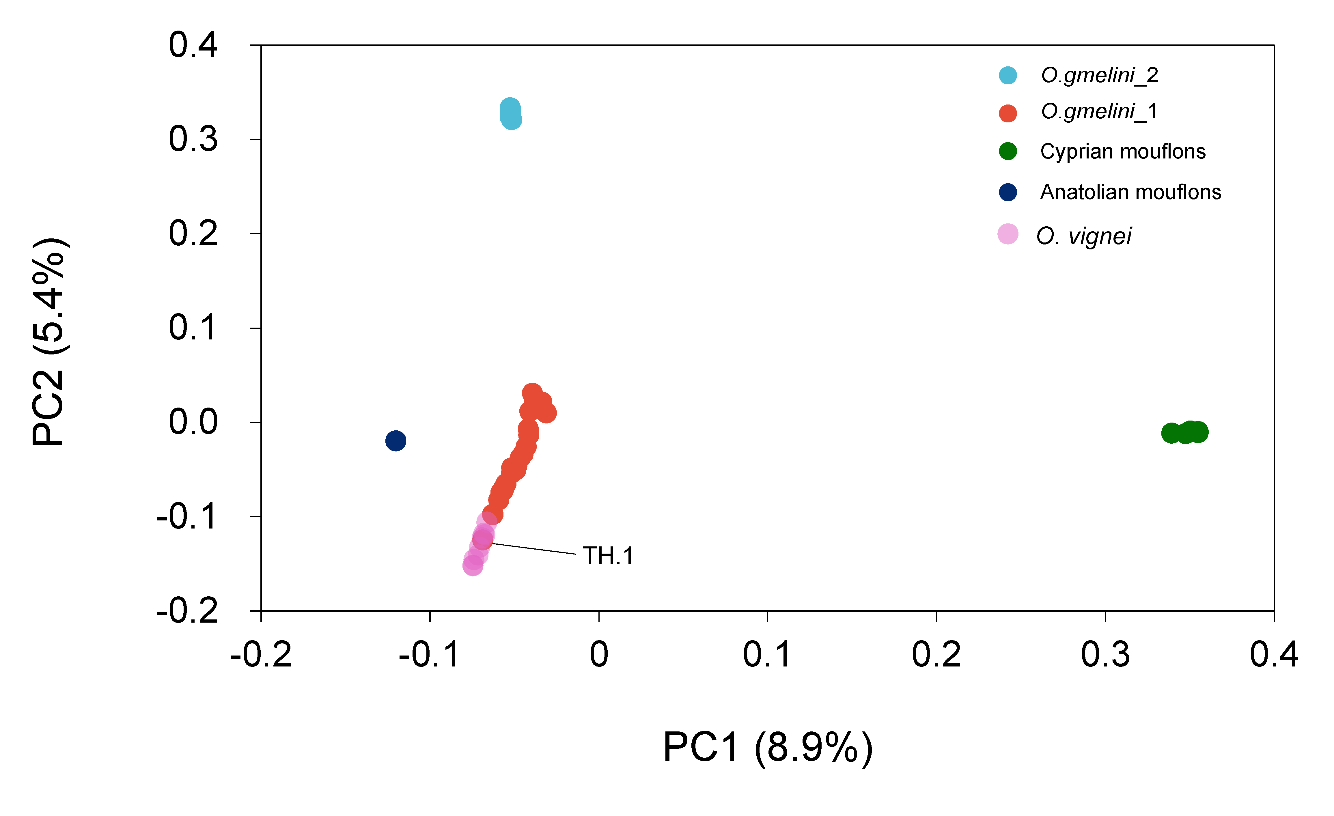
**

**Figure S5.** Neighbor-joining (NJ) trees of the Asiatic mouflon (7 Cyprian mouflons; 1 Anatolian mouflons; 7 *O. gmelini*_2; 23 *O.gmelini*_1) and domestic sheep (n = 738).

**
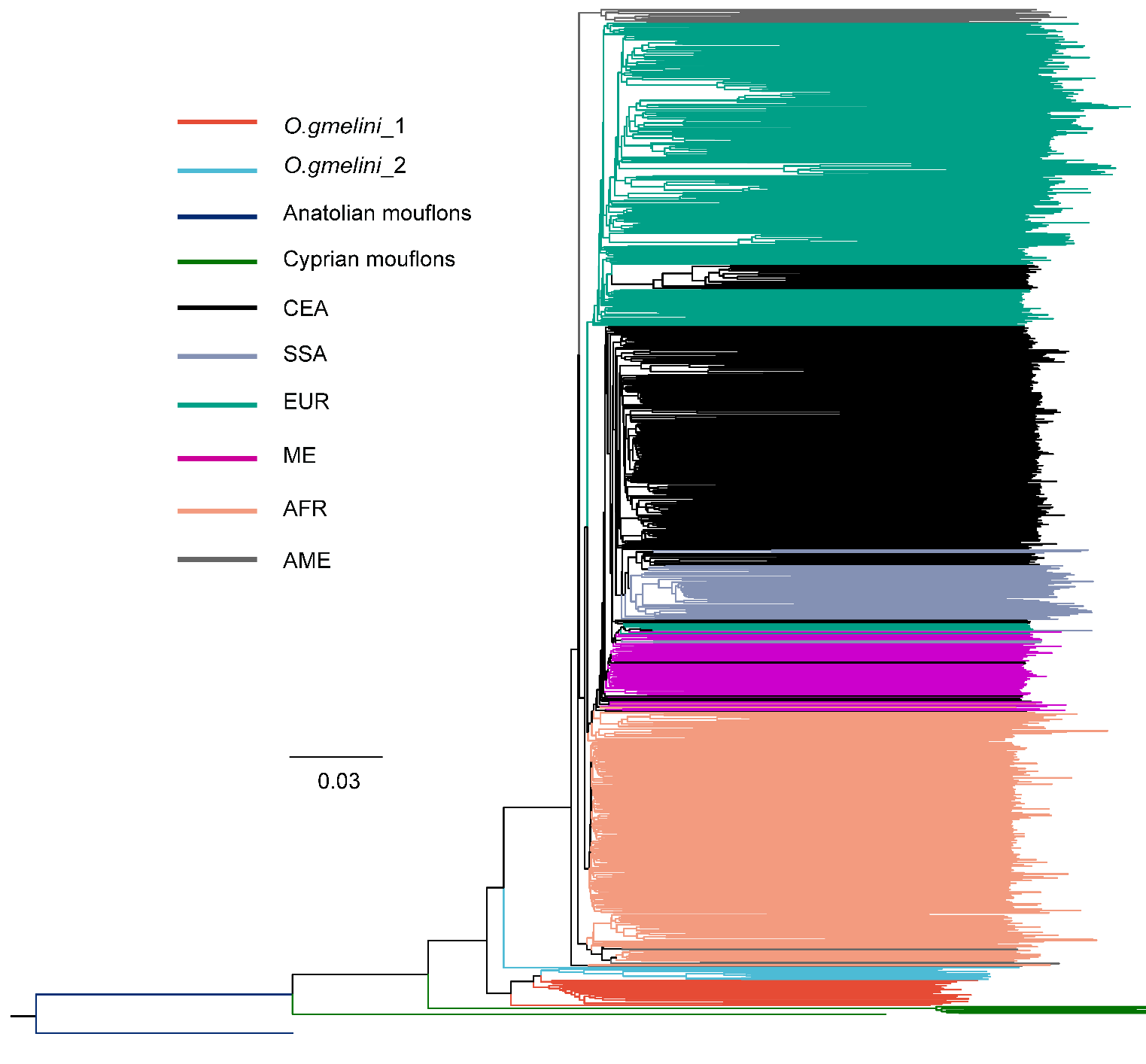
**

**Figure S6.**  Admixture of Asian mouflon and domestic sheep samples (7 Cyprian mouflons; 1 Anatolian mouflons; 7 *O. gmelini*_2; 23 *O.gmelini*_1; 738 domestic sheep) for *K*=2-9.


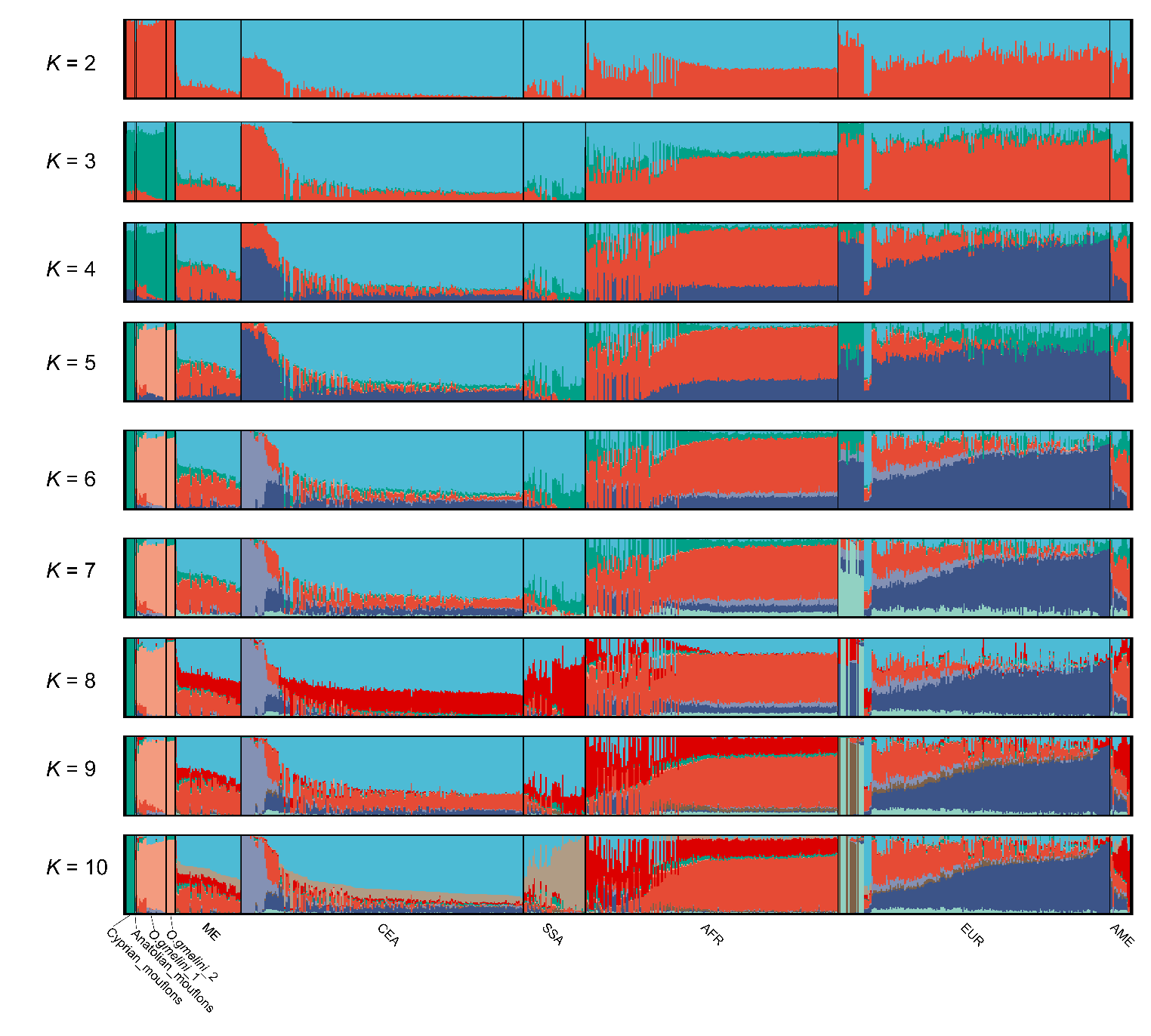


**Figure S7**. Maximum Likelihood Network Orientation reconstruction for the relationship between *O. gmelini*_1 and *O. gmelini*_2 using OrientAGraph. **a** migration event = 1; **b** migration events = 2; **c** migration events = 3; **d** migration events = 5.


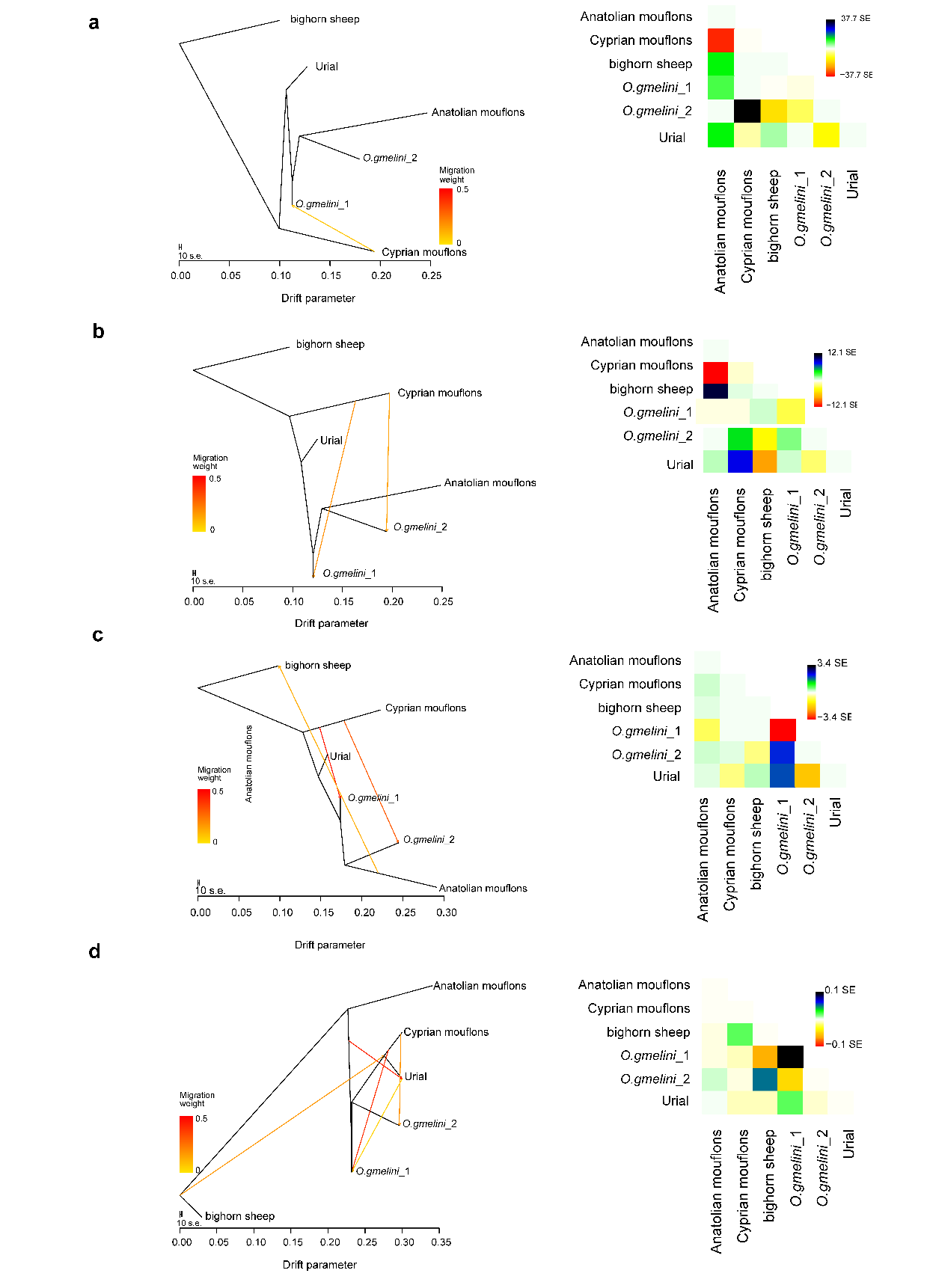


**Figure S8**. Admixture of SSA 18 breeds with two Iranian Asiatic mouflon populations for *K*= 2-9


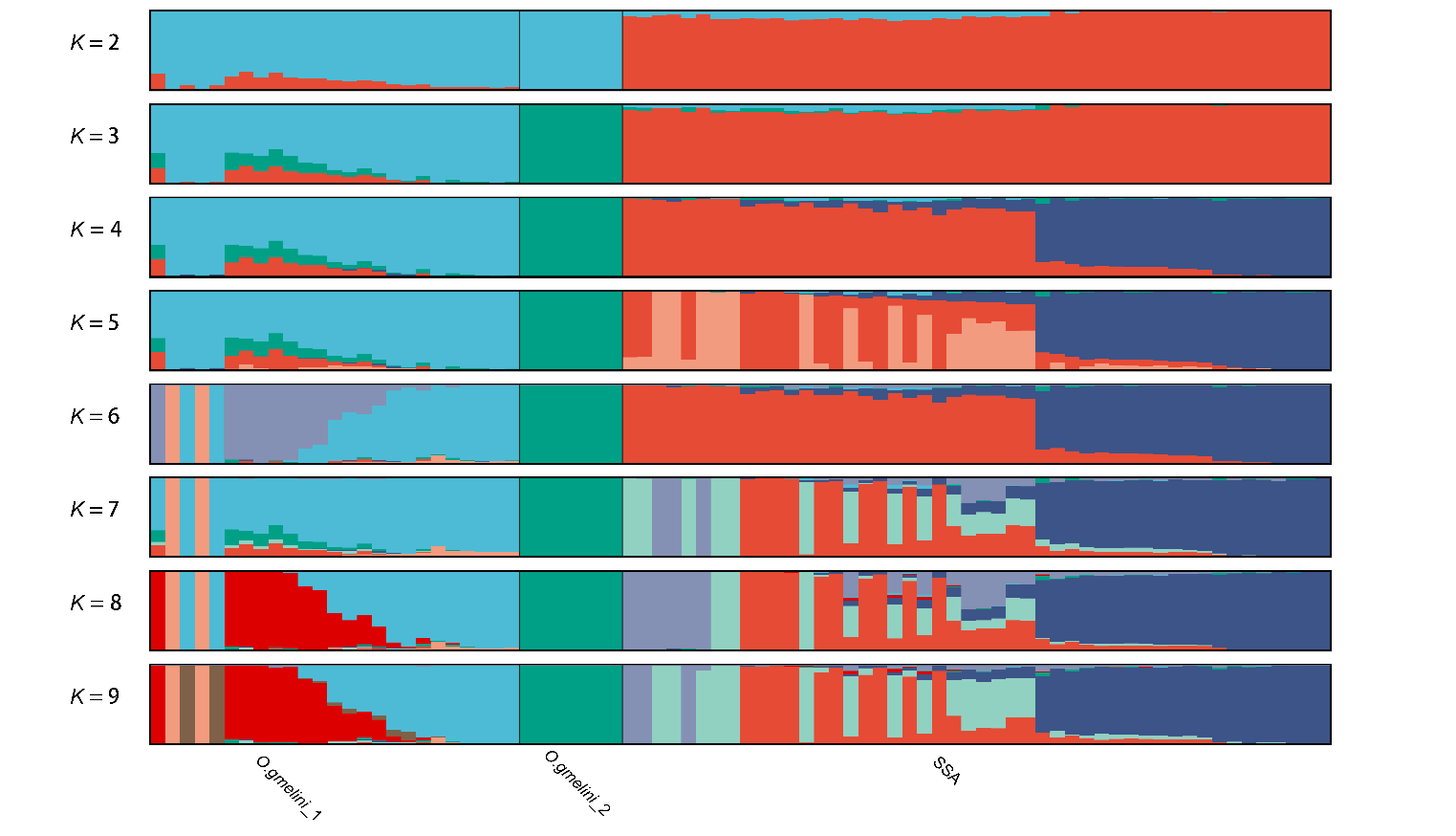


**Figure S9.** Word cloud summarizing QTL traits associated with the overlapping regions of *PBS* selection and introgression.


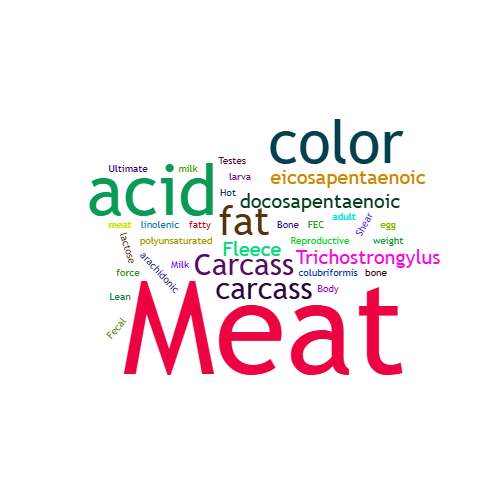

Supplement: Supplementary file 2 — Additional file 2: Figure S1. Title: Neighbor-joiningtree of wild and domestic sheep based on whole-genome SNPs. The tree showed the same two clusters of O. gmelini as Fig.1b and domestic sheep samples clustered based on their geographic distribution. Figure S2. PCA of wild and domestic sheep based on whole-genome SNPs. Figure S3. Neighbor-joiningtree of Asiatic mouflon based on whole-genome SNPs using bighorn sheep as the outgroup. Figure S4. PCA of Asiatic mouflon and urial based on whole-genome SNPs. Figure S5. Neighbor-joiningtree of the Asiatic mouflon and domestic sheep. Figure S6. Admixture of Asian mouflon and domestic sheep for K=2-9. Figure S7. Maximum Likelihood Network Orientation reconstruction for the relationship between O. gmelini_1 and O. gmelini_2 using OrientAGraph. a migration event = 1; b migration events = 2; c migration events = 3; d migration events = 5. Figure S8. Admixture of 18 SSA breeds with two Iranian Asiatic mouflon populations for K= 2-9. Figure S9. Word cloud summarizing QTL traits associated with the overlapping regions of PBS selection and introgression. [file 12711_2025_978_MOESM2_ESM.docx]
